# How common are depression and anxiety in adolescents with chronic fatigue syndrome (CFS) and how should we screen for these mental health co-morbidities? A clinical cohort study

**DOI:** 10.1007/s00787-020-01646-w

**Published:** 2020-09-22

**Authors:** Maria E. Loades, Rebecca Read, Lucie Smith, Nina T. Higson-Sweeney, Amanda Laffan, Paul Stallard, David Kessler, Esther Crawley

**Affiliations:** 1 Department of Psychology, University of Bath, Bath, BA2 7AY UK; 2 Bristol Medical School, University of Bristol, Bristol, UK; 3 Royal United Hospital, Bath, UK; 4 Department of Health, University of Bath, Bath, UK

**Keywords:** Prevalence, CFS, Mood, Depression, Anxiety, Screeni

## Abstract

Adolescents with Chronic Fatigue Syndrome/Myalgic Encephalomyelitis (CFS/ME) appear to be more likely to experience anxiety and/or depression using Patient Reported Outcome Measures (PROMs). However, we do not know how accurate these are at detecting problems in this patient group given the primary symptom of fatigue. We aimed to accurately determine the prevalence of anxiety/depression using gold-standard diagnostic interviews and evaluate the accuracy of PROMs measuring mood disorders in this patient group. We conducted a cross-sectional epidemiological study in a specialist tertiary paediatric CFS/ME service, England. The participants were164 12–18-year olds with clinician confirmed CFS/ME and their parents. The measures were a semi-structured diagnostic interview, the Kiddie Schedule for Affective Disorders and Schizophrenia, K-SADS, and questionnaires (Revised Children’s Anxiety and Depression Scale, RCADS; Spence Children’s Anxiety Scale, SCAS; Hospital Anxiety and Depression Scale, HADS). Parents completed the RCADS-P. 35% met the criteria for at least one common mental health problem. 20% had major depressive disorder, and 27% an anxiety disorder, with social anxiety and generalised anxiety being the most common. There was high co-morbidity, with 61% of those who were depressed also having at least one anxiety disorder. The questionnaires were moderately accurate (AUC > 0.7) at detecting clinically significant anxiety/depression, although only the RCADS-anxiety reached the predefined 0.8 sensitivity, 0.7 specificity target. Mental health problems are particularly common amongst adolescents with CFS/ME. Most screening tools were not sufficiently accurate in detecting clinically significant anxiety and depression, so these should be used with care in combination with thorough psychological/psychiatric assessment.

**Electronic supplementary material:**

The online version of this article (10.1007/s00787-020-01646-w) contains supplementary material, which is available to authorized users.

## Introduction

Chronic Fatigue Syndrome (CFS/ME) is common and disabling [1]. Around 30% of adolescents have high levels of depressive symptoms on standardised questionnaires [2–4]. A similar proportion have heightened anxiety, particularly social and separation anxiety [5]. However, as the symptoms of anxiety (e.g. restlessness) and depression (e.g. fatigue, sleep problems) overlap with those of CFS/ME, symptom conflation may lead to inaccurate estimates when relying on questionnaires. In addition, adolescents with CFS/ME do not feel questionnaires (Patient Reported Outcome Measures, PROMs) accurately capture their symptoms [6]. Some PROMs were designed for adults with no input from adolescents with CFS/ME [7]. It is; therefore, not clear whether the published prevalence rates are accurate or not. This could be resolved with diagnostic interviews. The existing studies using diagnostic interviews have also found high rates of anxiety disorders and depression, but are limited by small sample size and therefore, potential bias [8, 9] and are based on the previous versions of the diagnostic criteria.

The aim of this study was to (1) assess prevalence using diagnostic interviews with the current DSM-5 criteria in a large clinical cohort and (2) assess the accuracy of commonly used PROMs in detecting mental health problems in this population.

## Patients and methods

This was a cross-sectional study in a clinical cohort.

### Participants

Consecutively referred adolescents, age 12–18, with a clinician confirmed diagnosis of CFS/ME at their initial assessment at a specialist paediatric CFS/ME team within the National Health Service (NHS) in England were invited to participate. Recruitment spanned September 2016 to February 2019. Exclusion criteria were (1) having learning difficulties which precluded the completion of the study measures and (2) being unable to complete the diagnostic interview due to the functional impact of their CFS/ME or due to insufficient English ability. After 6 months, we adapted recruitment to include participants of the MAGENTA randomised control trial [10] (see Online Supplementary Materials Appendix 1 for further details).

### Measures

Self-reported age, sex, ethnicity and school attendance were collected routinely at clinical assessment.

*KSADS*—The Kiddie Schedule for Affective Disorders and Schizophrenia [11] is a semi-structured diagnostic interview for children and adolescents age 6–18. It is the gold-standard instrument for diagnosing depression [12, 13]. We focused on present psychiatric disorders (not previous, lifetime disorders). We used the following sections: MDD, panic disorder, agoraphobia, separation anxiety disorder, social anxiety disorder, phobic disorders, generalised anxiety disorder, obsessive–compulsive disorder, posttraumatic stress disorder, eating disorders and substance-related disorders [14]. We did not screen for: psychosis, conduct disorder, attention-deficit hyperactivity disorder, developmental disorders, enuresis and encopresis, nor did we screen for mania or disruptive mood dysregulation disorder which require historical information.

*RCADS—*The Revised Children’s Anxiety and Depression Scale [15] is made up of 47 items, of which 10 ask about depression. Respondents rate each item on a 0–3 scale and the total score is summed to give a depression score, where a higher score indicates greater depressive symptoms. Raw scores can be converted to age- and gender-adjusted *T* scores, which have a mean of 50 and a standard deviation of 10, using standardised tables. The RCADS has strong psychometric properties [16, 17] and good convergent validity with the KSADS [18]. Adolescents and parents completed the RCADS and RCADS-P, respectively. A briefer, 25 item versions of the RCADS and RCADS-P were also tested in the current study.

*HADS—*The Hospital Anxiety and Depression Scale [19] has 14 items: 7 depressive and 7 anxiety items. The HADS was designed to minimise overlap with physical disorders by excluding somatic symptoms. It has been validated for use with adolescents and has acceptable psychometric properties in this population [20]. Respondents rate items on a 0–3 scale, and the items for each subscale are summed to give a total score, with higher scores indicating more symptoms.

*SCAS*—The Spence Children’s Anxiety Scale [21, 22] is a 45-item self-report scale designed to measure symptoms relating to DSM-IV separation anxiety, social phobia, obsessive–compulsive disorder, panic-agoraphobia, generalised anxiety and fears of physical injury [23]. Each item is scored on a 0 (never)–3 (always) scale. The SCAS contains 7 positive filler questions, not included in the total score. The 38 anxiety items are summed, generating a total score out of a possible 114, with a higher score indicating greater anxiety. The SCAS has good psychometric properties [24].

*Fatigue—*CFQ: The Chalder Fatigue Questionnaire [25] has 11 items and responses are on a 0 (less than usual)–3 (much more than usual) scale. Responses are summed to create a total score. Higher scores indicate more fatigue. It has good reliability and validity in CFS samples [26] and has been used as an outcome measure adolescents with CFS [27, 28].

*Disability*—SF36-PFS: The 10 item Short Form-36 Physical Functioning Subscale [29] has three response options per item; 0 (yes limited a lot), 5 (yes, limited a little), and 10 (no, not limited at all). Responses are summed, resulting in a total score out of a possible 100. Higher scores indicate better physical functioning. The psychometric properties of this scale are acceptable in adolescents with CFS [30].

### Ethical Approval

Ethical approval was granted by the NHS Health Research Authority (16/SW/036) and the Department of Psychology at the University of Bath (16–203). The MAGENTA trial was approved by the NHS Health Research Authority (15/SW/0124).

### Procedure

Potential participants were provided with a participant information sheet at their initial clinical assessment and offered the opportunity to agree to be contacted by the research team via telephone or email. Interested participants were provided with further information, and we subsequently obtained verbal consent and written consent, either online via REDCap (research electronic data capture [31]) or pen-and-paper. For participants who were < 16 years old, parental consent and adolescent assent were obtained.

Those participants recruited within the MAGENTA trial were assessed for eligibility for the trial by their assessing clinician (eligibility criteria are detailed in the study protocol [10]). Eligible patients who consented to be contacted were subsequently given further information about the study by a research nurse over the telephone, who obtained consent/assent via REDCap. From February 2017, this included consenting to participate in the KSADS interview for those participants of the MAGENTA trial who were ≥ 12 years old.

Participants provided consent to use data collected from the following clinical PROMs completed prior to first clinical assessment: SCAS, HADS, the CFQ and the SF36PFS. After recruitment, participants completed the RCADS. Participants interviewed as part of the MAGENTA trial did not complete the RCADS.

The KSADS interview was conducted via Skype (*N* = 48, 29.3%), telephone (*N* = 89, 54.3%), at the hospital site (*N* = 13, 7.9%) or the participant’s home (*N* = 10, 6.1%), according to participant preference. The KSADS has been administered by telephone in other studies [32, 33]and diagnostic interviews have been administered via videoconferencing and telephone in chronic illness samples to minimise participant burden [34]. Participants were given the option of being interviewed with a parent present. If they opted to be interviewed alone (*N* = 31, 18.9%), the parent was briefly interviewed separately. The questions asked of parents when interviewed alone were focused on the more observable, behavioural aspects of depression and anxiety, such as irritability, and were also informed by the areas that the adolescent interview had highlighted. Parents were invited to add any other concerns they had. The resultant information was integrated into a summary judgement by the interviewer; due to the internalising nature of depression and anxiety, adolescent report given more weight when there were discrepancies between self- and parent-report. Research assistants were trained to accommodate the needs of young people with CFS/ME; interviews were kept brief, participants could take a rest breaks whenever they wished and the interview could be conducted over several sessions within the same week where needed. Interviews lasted between 9 and 199 min in total (mean 44.43, SD 28.39).

Interviews were conducted by research assistants (psychology students) who had completed a two-day training course on administration. Specific emphasis was on training the interviewers how to ask about each symptom screened for on the KSADS in a manner appropriate to the context of CFS/ME. For example, when asking about anhedonia, questions were asked about what activities adolescents engaged in currently that gave them enjoyment, even if they were unable to do the enjoyable activities they usually would. Similarly, when questions about fatigue particularly emphasised the link between fatigue and other mood relevant symptoms and experiences.

Research assistants were subsequently closely supervised, including of audio recordings of interviews. Following each interview, the interviewer made a provisional diagnostic conclusion, which was reviewed and discussed with an experienced mental health professional (ML) for all cases. ML attended a 2-day training course on KSADS administration at the outset of the study, including taking part in inter-rater reliability exercises. In addition, 23 (14%) interviews were checked by a second interviewer, blind to the conclusion made by the interviewer. Both raters agreed on 195 of 207 (94.2%) diagnostic decisions, which is considered fair agreement (Cohen 1960),* κ* = 0.20 *p* = 0.007, 95% CI [− 0.16, 0.56].

### Data analysis

The data were analysed using IBM SPSS Statistics 23 and MedCalc. Participants were classified as having DSM-5 defined MDD, panic, agoraphobia, separation anxiety, social anxiety, phobic, generalised anxiety, obsessive compulsive or posttraumatic stress disorder based on the KSADS interview. Descriptive statistics were analysed to determine the prevalence of anxiety disorders and MDD.

The diagnostic interview, the KSADS, was the gold standard against which the questionnaire scores were compared. Receiver-operating characteristic (ROC) curves were constructed using the DeLong method and the diagnostic accuracy of the RCADS-D, RCADS-any anxiety disorder, SCAS, HADS-D and HADS-A was quantified using the area under the ROC curve (AUC). An AUC of ≥ 0.7 was assumed to indicate at least moderate accuracy in identifying those who have depression/anxiety. For clinical purposes, sensitivity was considered more desirable than specificity; therefore, we sought the optimal threshold which had a sensitivity of ≥ 0.8 and specificity of ≥ 0.7.

## Results

**Fig. 1:**
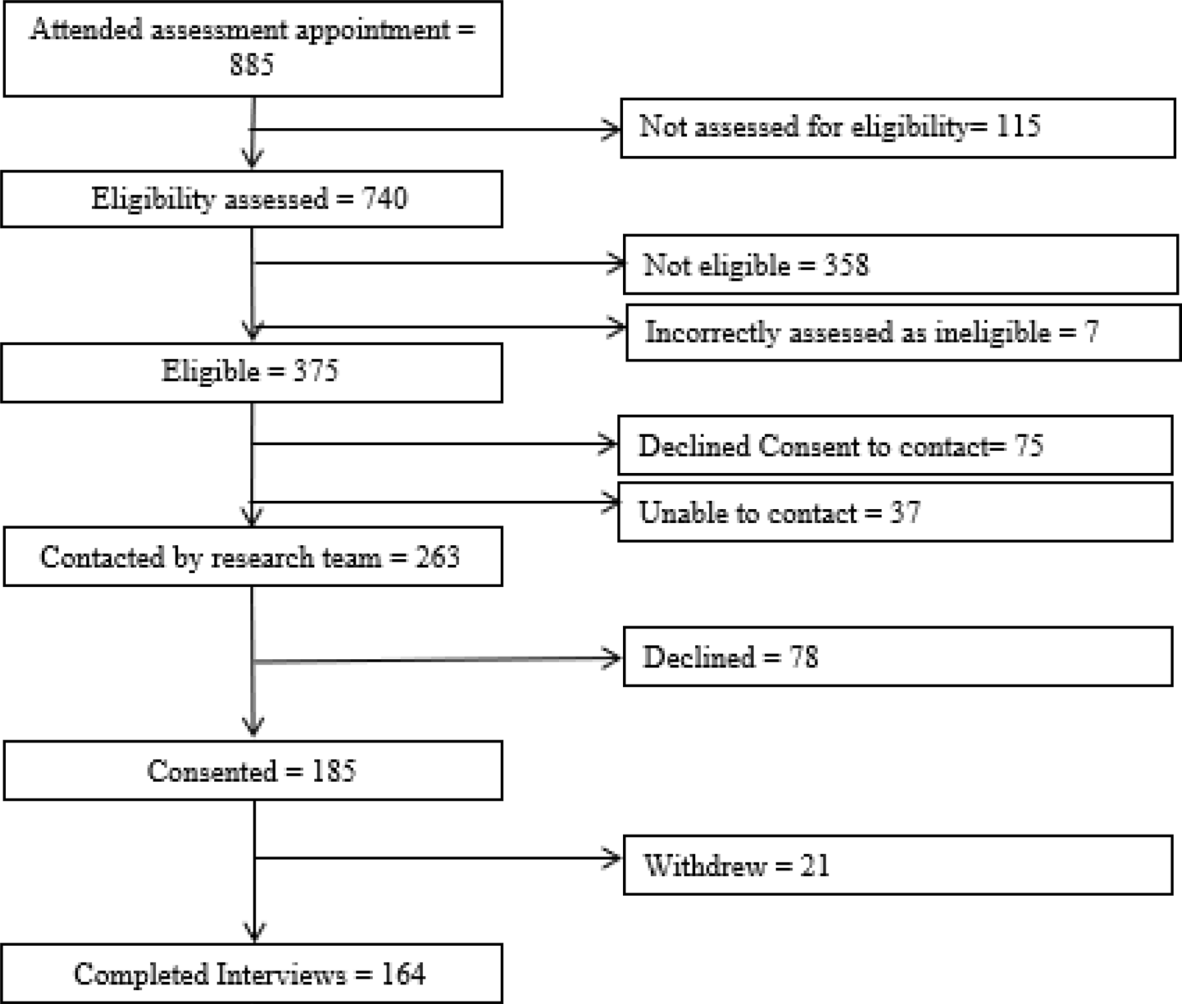
Consort diagram showing number of participants at each stage of recruitment

Of the 375 patients who attended an initial clinical assessment appointment and were assessed as meeting the eligibility criteria, 263 (70.1%) agreed to further contact from the research team and 185 (70.3%) of these consented to participate in the study, with 21 subsequently withdrawing (see Fig. 1). Those who were recruited were similar to those who were not recruited (i.e. eligible, but not offered the opportunity to participate by clinicians or declined consent to contact, see Supplementary Information Table S1). Of the 164 patients (43.7% of those eligible) interviewed, most were females (70.1%). The mean age was 14.99 years, mainly White British participants (see Table 1). Only 13 (7.9%) participants were attending school full time, with 67 (40.8%) participants attending less than 40% of the time and the participant mean score on the SF36PFS as a measure of disability was 51.66, which is comparable to other cohort studies in the same setting [2, 27, 35] (see Supplementary Materials Table S2). In almost all cases, a parent was interviewed as well as the adolescent themselves (*N* = 156, 95%). The majority (*N* = 140) were mothers. In 9 instances, fathers were interviewed and both parents in seven instances.

**Table 1:** Characteristics of the sample

Gender	
Male	49 (29.9%)
Female	115 (70.1%)
Ethnic origin	
White British	146 (89.0%)
Unspecified	10 (6.1%)
Other White	6 (3.7%)
Other British	1 (0.6%)
Pakistani	1 (0.6%)

	Range	Mean (SD)	Cronbach’s *α*
Age (years)	12–18	14.99 (1.50)	
SCAS total	3–99	34.44 (17.97)	0.93
HADS-D total	0–18	7.85 (3.76)	0.76
RCADS-A	5–78	35.28 (20.73)	0.96
RCADS-D	2–27	15.02 (5.38)	0.84
CFQ	11–33	25.25 (4.47)	0.83
SF36PFS	0–100	51.66 (24.04)	0.90

*CFQ* Chalder Fatigue Questionnaire, *HADS-A* Hospital Anxiety and Depression Scale-Anxiety, *HADS-D* Hospital Anxiety and Depression Scale-Depression, *RCADS-A* Revised Children’s Anxiety and Depression Scale-Any Anxiety, *RCADS-D* Revised Children’s Anxiety and Depression Scale-Depression, *SCAS* Spence Children’s Anxiety Scale, *SF36PFS* Short Form 36 Physical Functioning Subscale

Out of the 164 participants recruited, 58 (35.4%) met the criteria for at least one mental health diagnosis of whom 13 (7.9%) had major depressive disorder (MDD) only, 25 (15.2%) had an anxiety disorder only. Approximately a third (20, 34.5%) had both anxiety and depression which means that 33 participants (20%, 95% CI 13.1–27.1) met the criteria for MDD and 45 participants (27.4%, 95% CI 20.4–34.4) met the criteria for at least one anxiety disorder (see Table 2).

**Table 2:** Number of participants meeting DSM-5 diagnostic criteria on the KSADS

Disorder	Participants who received diagnosis—*N* (%)
Major depressive disorder	33 (20.1)
Any anxiety disorder	45 (27.4)
Panic	6 (3.7)
Agoraphobia	4 (2.4)
Separation anxiety	1 (0.6)
Social anxiety	19 (11.6)
Phobia	14 (8.5)
Generalised anxiety disorder (GAD)	17 (10.4)
Obsessive compulsive disorder (OCD)^a^	3 (1.8)
Eating disorder	0
Post-traumatic stress disorder (PTSD)^a^	1 (0.6)

^a^OCD was included as an anxiety disorder, but PTSD was not, as this is consistent with the PROMs used

Consistent with the predominance of females in the sample, more females than males were diagnosed with depression (*N* = 23, 69.7% female) and anxiety (*N* = 34, 75.6%). The mean age of those meeting diagnostic criteria on the KSADS was slightly higher than the overall sample mean, but not significantly so (depression: mean difference = 0.22, 95% CI − 0.34–0.78, *p* = 0.435; Anxiety: mean difference = 0.41, 95% CI − 0.08–0.90, *p* = 0.101).

The most prevalent anxiety disorder types were social anxiety disorder (*N* = 19, 11.6%) and generalised anxiety disorder (*N* = 17, 10.4%). Fourteen (8.5%) had a phobic disorder with 4 participants meeting the criteria for an animal phobia, 4 natural environment phobias (e.g. thunderstorms, heights), 1 blood/injection/injury phobia and 5 situational type phobias (e.g. planes, elevators). Only one participant met the full diagnostic criteria for separation anxiety disorder, although a further 16 (9.8%) presented with subthreshold symptoms of separation anxiety. Thirty participants met the criteria for only one anxiety disorder diagnosis, with 11 meeting criteria for two anxiety disorders and 4 meeting the criteria for three anxiety disorders.

Of the 33 diagnosed with co-morbid depression, the vast majority (*N* = 30, 90.9%) had significant fatigue which they perceived to be linked to and exacerbated by their mood, over and above fatigue from their CFS/ME. Furthermore, cognitive difficulties were fairly pervasive (*N* = 29, 87.9%). Most had depressed mood (*N* = 23, 69.7%) and/or psychomotor retardation or agitation (*N* = 23, 69.7%). Many also had difficulties sleeping (*N* = 21, 63.6%) and/or irritability (*N* = 21, 63.6%). Relatively few (*N* = 6, 18.2%) had suicidal thinking or feelings of worthlessness (*N* = 13, 39.4%). Anhedonia was described in just over 50% of the sample (see Table 3).

**Table 3:** Frequency of threshold symptoms of depression on the KSADS in those diagnosed with Major depressive disorder (MDD)—data shown as *N* (%)

DSM symptom	Yes
Depressed mood	23 (69.7)
Irritable mood	21 (63.6)
Anhedonia	18 (54.5)
Appetite/weight change	17 (51.5)
Insomnia/hypersomnia	21 (63.6)
Psychomotor agitation/retardation	23 (69.7)
Fatigue^a^	30 (90.9)
Feelings of worthlessness/excessive or inappropriate guilt	13 (39.4)
Reduced concentration/slowed thinking/indecisiveness	29 (87.9)
Recurrent thoughts of death/suicidal ideation/suicide attempt	6 (18.2)

^a^Fatigue was scored as threshold only if it was perceived to be linked to mood by (1) becoming more prominent at the onset of the low mood episode and/or (2) tending to be worse when mood was worse

**Fig. 2:**
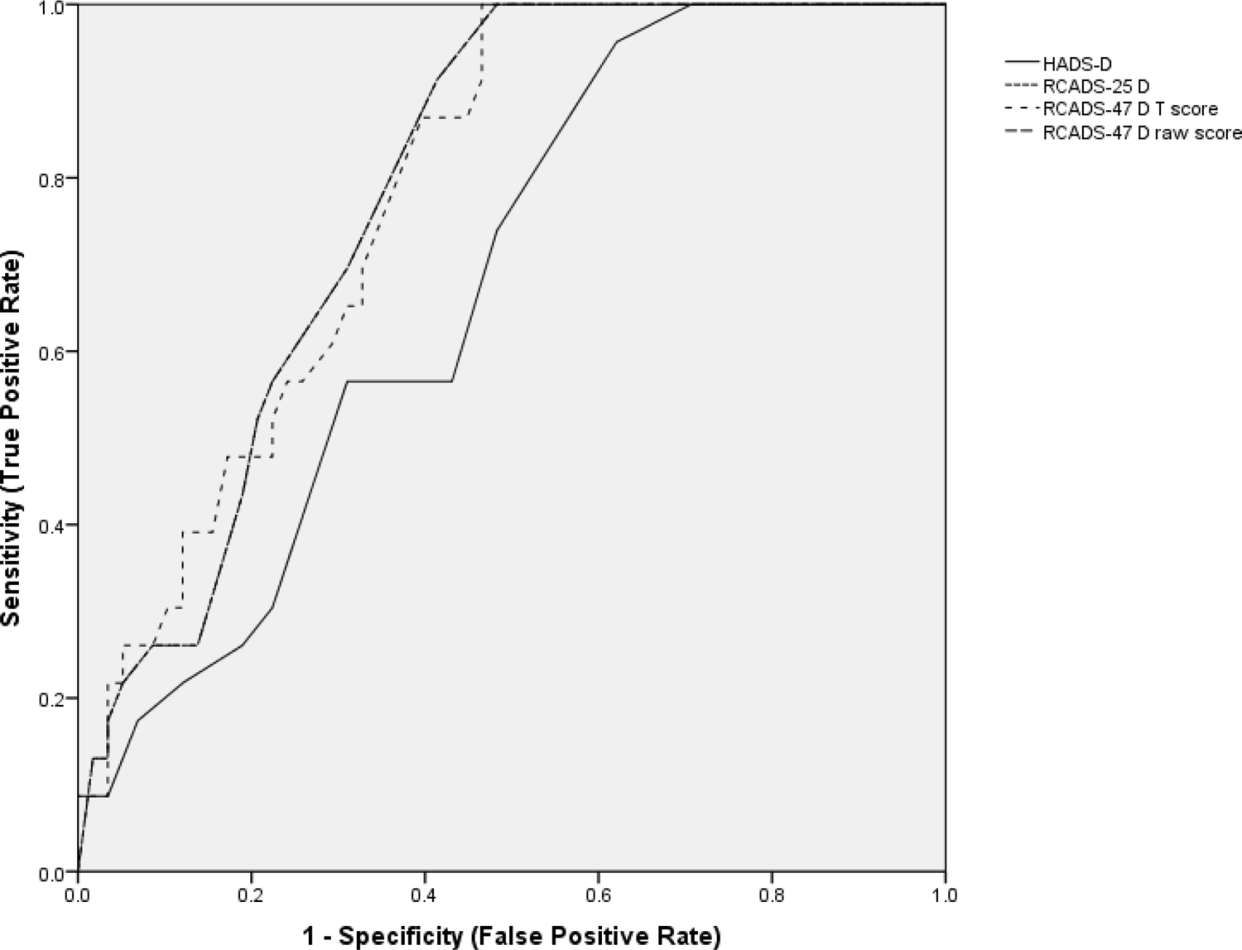
Receiver operating curves for depression measures

**Fig. 3:**
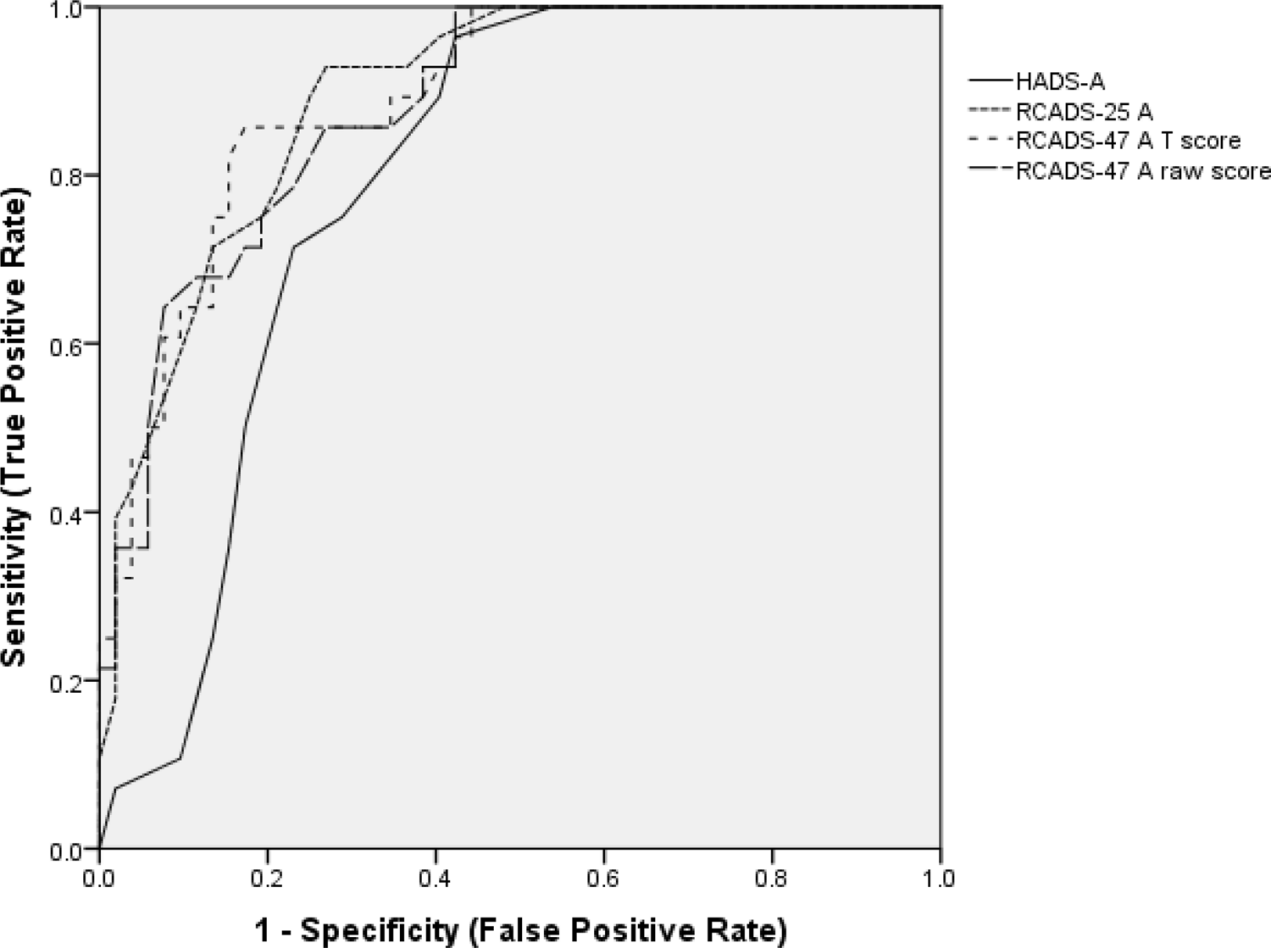
Receiver operating curves for anxiety measures

All PROMs (i.e. the RCADS, HADS and SCAS) scored > 0.7 for area under the curve, AUC, indicating at least moderate accuracy (see Table 4 and Figs. 2 and 3). The RCADS-A performed the best and both the brief and full version were similar with little difference between the raw scores and the age- and gender-adjusted T scores. For all versions of the RCADS-A, we identified threshold scores reaching the required ≥ 0.8 sensitivity and ≥ 0.7 specificity thresholds. We could not identify scores on the HADS-A and SCAS which reached these thresholds (see Table 4). None of the depression measures were sufficiently accurate to reach the 0.8/0.7 requirement (see Table 4).

**Table 4:** ROC analysis for PROMs

	Measure	AUC	SE of AUC	95% CI for AUC	*Z* statistic for AUC	Optimum threshold for diagnosis	Sensitivity	Specificity
Adolescent self-reported measures								
Depression (MDD)	RCADS-D raw score	0.789	0.0464	0.690–0.869	6.238	> 14 > 15	92.00 72.00	59.38 68.75
	RCADS-D *t* score	0.787	0.0485	0.685–0.869	5.929	> 68 > 69	80.00 72.00	63.33 66.67
	HADS-D	0.714	0.0425	0.636–0.783	5.032	> 7 > 8	80.65 67.74	55.20 61.60
Anxiety (any anxiety disorder diagnosis)	RCADS-A (37 item) raw score	0.879	0.0357	0.792–0.938	10.602	**> 37** **> 38**	**86.67** **80.00**	**72.88** **76.27**
	RCADS-A (37 item) *t* score	0.889	0.0347	0.802–0.947	11.221	**> 55** **> 56**	**86.67** **86.67**	**80** **81.82**
	RCADS-A (15 item) raw score	0.890	0.0337	0.806–0.946	11.582	**> 10** **> 11** **> 12**	**93.33** **90.00** **80**	**72.88** **74.58** **77.97**
	RCADS-A (15 item) *t* score	0.889	0.0347	0.802–0.947	11.221	**> 56**	**86.67**	**81.82**
	HADS-A	0.774	0.0403	0.700–0.837	6.795	> 9 > 10	83.33 71.43	60.18 68.14
	SCAS	0.744	0.0449	0.665–0.814	5.438	> 37 > 39	76.32 71.05	70.48 76.19
Parent informant-reported measures								
Depression (MDD)	RCADSP-D raw score	0.780	0.0517	0.688–0.869	5.582	> 15	69.6	79.7
	RCADSP D *t* score	0.787	0.0485	0.685–0.869	5.929	> 68	80	63.33
Anxiety (any anxiety disorder diagnosis)	RCADSP-A (37 item)	0.858	0.0417	0.765–0.924	8.565	**> 31**	**83.33**	**76.36**
	RCADSP-A (37 item) *t* score	0.858	0.0438	0.762–0.925	8.162	** > 62** **> 64**	**83.33** **80.00**	**78.43** **80.39**
	RCADSP-A (15 item)	0.780	0.0517	0.765–0.922	8.449	**> 9**	**80.00**	**75.44**
	RCADSP-A (15 item) *t* score	0.861	0.0421	0.767–0.928	8.572	**> 58**	**80.00**	**75.00**

Bold indicates that the minimum required 0.8 sensitivity/0.7 specificity criterion is met by this threshold score

## Discussion

This is the first large clinical cohort study to accurately measure both anxiety and depression using diagnostic interviews applying DSM-5 criteria in adolescents with CFS/ME. We found that 1 in 3 met the diagnostic criteria for a common mental health disorder. The majority of those who met the criteria for a mental health problem had both anxiety and depression; 20% of adolescents attending a specialist CFS/ME service meet the criteria for Major Depressive Disorder (MDD) and 27% the criteria for at least one anxiety disorder. Some met the criteria for up to three co-occurring anxiety disorders. The high co-morbidity in this group suggests that if one is diagnosed, the other needs to be screened for. Unlike in other conditions, the PROMs were not sufficiently accurate, apart from the RCADS-A (self-report and parent versions) and even using the optimum cutoffs that we identified will likely result in false positives and false negatives.

Consistent with the epidemiology of CFS/ME [36], we recruited more females than males. Despite the recruitment difficulties encountered [37], the participants recruited to this study were comparable to those recruited in other cohort studies and randomised controlled trials in the same service setting (see Supplementary Materials Table S3).

This study is consistent with the previous work, which shows that depression and anxiety are higher in adolescents with CFS/ME [5, 8, 9, 38] than in healthy populations [39, 40]. The high rates of depression and anxiety seen in adolescents with CFS/ME could be due to a variety of factors including social loss [41–43], the inability to take part in activities [41, 42] or other underlying causal factors including shared genetic vulnerability [44] and previous trauma [45, 46]. Alternatively, a tendency to avoid anxiety-provoking situations, such as phobic stimuli or physical activity following a viral infection, could independently contribute to both anxiety and CFS/ME, for instance [47, 48].

We found high rates of anxiety disorders. This is particularly striking given that the KSADS applies stricter criteria than the Anxiety Diagnostic Interview Schedule (ADIS), which is most commonly used in anxiety studies [49]. We did not show the high rates of separation anxiety found in previous work [5]. This may be because previous studies recruited younger participants, more likely to be experiencing separation anxiety disorder [50]. Alternatively, it may be because of the reliance of previous studies on questionnaires, which meant that they were unable to distinguish between those with raised separation fears (which were common in our sample) and those meeting the full diagnostic criteria for separation anxiety disorder. The level of specific phobia was comparable to general population samples of adolescents [51, 52]. The high levels of Generalised Anxiety Disorder are a new finding. This may be because of a general tendency to avoid anxiety-provoking situations [47, 48] or shared vulnerability [53].

In those adolescents who were depressed, the pattern of symptoms was different to that commonly found in samples recruited via Child and Adolescent Mental Health Services [54, 55]. Rates of suicidal ideation and feelings of worthlessness were particularly low, which may be due to referral criteria for different specialist services, with those who report suicidal ideation more likely to be referred to mental health services. Rates of somatic symptoms including fatigue and cognitive difficulties were extensive in our sample, which are also common in adolescents with CFS/ME [35] and may be compounded in those who have co-morbid depression. It may also be that there are different subtypes of depression, including an inflammation-linked somatic subtype, which is more frequent in CFS/ME [56]. Our findings suggest that depression in adolescents CFS/ME may be characterised less by negative self-perceptions and self-injurious behaviours and more by anhedonia and somatic symptoms.

Whilst this is the first study to accurately define the prevalence of both anxiety and depression in CFS/ME, the high rate of comorbidity between the two is perhaps not surprising given the co-occurrence in healthy adolescents [57]. It does; however, mean that clinicians and clinical services ought to screen for both mood and anxiety disorders when planning treatment.

As the KSADS diagnostic interview is too resource intensive for use in most routine clinical care settings, a screening measure that is sufficiently accurate and appropriate for the population is urgently needed. Apart from the RCADS anxiety, the PROMs we examined did not meet our pre-specified criteria for accuracy. It is encouraging that both the self- and parent-report versions of the RCADS anxiety subscale (37 items) and the brief versions of this (15 items) did meet these criteria. In CFS/ME, reducing burden on patients is of utmost importance and using the brief version (although this is at the cost of information about specific anxiety disorder types) and using parent informants where adolescents are too unwell to complete the questionnaire themselves could be useful. The RCADS-depression subscale did not meet the criteria for accuracy. Furthermore, the HADS did not meet the required accuracy criteria for either depression or anxiety; we would therefore recommend that services use other PROMs such as the RCADS for anxiety. The HADS was developed for adults and then validated for use in adolescents, whilst the RCADS was specifically developed for children and adolescents and may therefore be more developmentally appropriate. A more accurate self-report measure of depression symptoms among adolescents with CFS/ME is needed. The Mood and Feelings Questionnaire [58] which is the gold standard questionnaire for screening for depression in adolescents [59] and may overlap less with the core symptoms of CFS/ME could be worth investigating.

### Strengths and limitations

This is a considerably larger study than has previously been undertaken in adolescents with CFS/ME. However, we did not include under 12s and cannot make any conclusions about anxiety and depression in children. We used diagnostic interviews, assessed the latest DSM-5 criteria and aimed to recruit all eligible patients seen within the largest specialist paediatric CFS/ME clinic in the UK. Whilst our sample is large, many patients declined to participate so our sample may not be fully representative of the population seen in this clinic or wider services. Furthermore, our findings may not apply to settings other than specialist services. Finally, whilst the KSADS is the best diagnostic instrument for diagnosing depression, it may not always be 100%. Accuracy depends on the diagnostic judgements by trained raters. This needs to be acknowledged, but we minimised this possibility through training and individual case review.

## Conclusion

A substantial minority of adolescents with CFS/ME meet diagnostic criteria for a co-morbid major depressive disorder or an anxiety disorder (generalised anxiety, social anxiety and specific phobia are most common) or both. Identifying patients who are experiencing mental health problems is key to treatment for both CFS/ME and psychological distress and to enabling recovery and maximising functioning. Clinicians need to be aware that symptoms typically associated with depression (such as suicidal thinking) and anxiety are not necessarily present in adolescents with CFS/ME. Many patients have both anxiety and depression; therefore, clinicians should screen for one if they find the other. A full clinical assessment should be used alongside the RCADS questionnaire. Clinicians in paediatric settings may benefit from additional training in identifying mental health problems in adolescents with CFS/ME specifically. A combination of qualitative and longitudinal epidemiological research would shed further light on the factors which contribute to anxiety amongst this population.

## Electronic supplementary material

Below is the link to the electronic supplementary material.

Supplementary file1 (DOCX 49 kb)

## Abbreviations

CFS/ME: Chronic Fatigue Syndrome/Myalgic Encephalomyelitis
DSM-5: Diagnostic and Statistical Manual of Mental Disorders version 5
GAD: Generalised anxiety disorder
KSADS: Kiddie schedule for affective disorders and schizophrenia
MDD: Major depressive disorder
NHS: National Health Service
OCD: Obsessive–compulsive disorder
PTSD: Post-traumatic stress disorder
CFQ: Chalder Fatigue Questionnaire
HADS-A: Hospital Anxiety and Depression Scale-Anxiety
HADS-D: Hospital Anxiety and Depression Scale-Depression
RCADS-A: Revised Children’s Anxiety and Depression Scale-Any Anxiety
RCADS-D: Revised Children’s Anxiety and Depression Scale-Depression
SCAS: Spence Children’s Anxiety Scale
SF36PFS: Short Form 36 Physical Functioning Subscale
