# Supplementary material for: How common are depression and anxiety in adolescents with chronic fatigue syndrome (CFS) and how should we screen for these mental health co-morbidities? A clinical cohort study
Source: Eur Child Adolesc Psychiatry. 2020 Sep 22;30(11):1733–43. doi: 10.1007/s00787-020-01646-w (PMC8558286; doi:10.1007/s00787-020-01646-w)
Supplement: Supplementary file 1 — Supplementary file1 (DOCX 49 kb) [file 787_2020_1646_MOESM1_ESM.docx]

**Table S1.** Comparison of questionnaire scores (Mean, S.D.) for participants (N = 164) and non-consenting eligible patients who completed routine questionnaires prior to their initial clinical assessment (N = 123)

| **Measure** | **Depression (HADS-D)** | | **Anxiety (HADS-A)** | | **Physical Functioning (SF36PFS)** | |
| --- | --- | --- | --- | --- | --- | --- |
|  | Participated | Eligible, did not participate | Participated | Eligible, did not participate | Participated | Eligible, did not participate |
| Total N  Mean (S.D) | N = 164  7.85 (3.76) | N = 123  8.57 (4.54) | N = 164  9.15 (4.29) | N = 123  10.11 (5.04) | N = 164  51.66 (24.04) | N = 123  49.18 (25.97) |
|  | Mean difference = 0.72 (95% CI -0.25-1.69),  p = 0.143 | | Mean difference = 0.96 (95% CI = -0.13-2.05), p = 0.083 | | Mean difference = -2.48 (95% CI = -8.32-3.36), p = 0.404 | |

Note: Research Ethics Committee approval was granted for using the data from non-consenting eligible participants for this purpose.

**Table S2.** School attendance

| **Self-reported attendance** | **N (%)** |
| --- | --- |
| None | 22 (13.4) |
| 10% | 13 (7.9) |
| 20% | 7 (4.3) |
| 40% | 25 (15.2) |
| 60% | 25 (15.2) |
| 80% | 38 (23.2) |
| Full time | 13 (7.9) |
| Not applicable | 5 (3.1) |
| Missing | 16 (9.8) |

**Table S3.** Comparison of current study cohort to other cohorts recruited at the same recruitment site

| **Study** | **Crawley & Sterne (2009)^1^** | **Crawley, Hunt & Stallard (2009)^2,3^** | **Bould et al (2013)** | **Collin et al (2015)^4^ UK vs Dutch cohort** | **Crawley et al (2017)^5^ SMC group** | **Crawley et al (2017)^5^ SMC + LP group** | **Current Study** |
| --- | --- | --- | --- | --- | --- | --- | --- |
| Recruitment window | 2004-2007 | 2004-2007 | 2005-2011 | 2004-2014 | 2010-2013` | 2010-2013 | 2016-2019 |
| Ages included | < 18 y/os | 7-17 | 12-18 | 12-18 | 12-18 | 12-18 | 12-18 |
| N | 211 | 159 | 542 | ~1400 | 49 | 52 | 164 |
| Mean (S.D.) unless otherwise specified | | | | | | | |
| Age | Median 14.6 | Median 14.2 (IQR 12.5-15.7) | 14.8 (1.5) | 15.0 (1.8) | 14.5 (1.6) | 14.7 (1.4) | 14.99 (1.50) |
| SF36PFS | Median 20 (IQR 16-23) |  | 51.0 (24.4) | Median 50 (IQR 33-70) | 56.0 (21.5) | 53.0 (18.8) | 51.66 (24.04) |
| CFQ | Median 25 (IQR 20-28) | 23.9 (5.5) | 24.9 (4.9) | Median 25 (IQR 22-29) | 25.1 (4.2) | 25.0 (4.2) | 25.25 (4.47) |
| School attendance ≤ 40% | N = 120 (56.9%) |  | N = 214 (41.2%) |  | N = 25 (51.0%) | N = 22 (44.0%) | N = 67 (40.9%) |
| HADS-D |  |  | 7.6 (3.8) |  | 8.1 (4.4) | 7.5 (3.1) | 7.85 (3.76) |
| HADS-A |  |  |  |  | 10.4 (4.4) | 8.8 (4.5) | 9.15 (4.29) |
| SCAS |  | 29.7 (S.D. not stated) |  |  | 40.3 (20.1) |  | 34.44 (17.97) |

**Appendix 1. Further Details of Study Recruitment**

During the first 6 months of recruitment, patients who agreed to participate in a concurrent randomised controlled trial, the Managed Activity Graded Exercise iN Teenagers and pre-Adolescents (MAGENTA) trial ^6^, were excluded from the current study. Subsequently, to ensure representativeness and therefore enable a true estimate of prevalence, these participants were also included in the current study as the diagnostic interview was administered as part of the baseline trial procedures (see figure S1). The MAGENTA trial was approved by the NHS Health Research Authority (15/SW/0124) and an amendment was approved to include the KSADS as part of baseline trial procedures for those age ≥ 12 from March, 2017 onwards.

Figure S1. Diagram showing recruitment routes into the study

Complete postal assessment form including HADS & SCAS and attend initial clinical assessment appointment

Eligibility assessed for MAGENTA

Inclusion criteria:

- Age 8 - 17
- CFS/ME diagnosis

Exclusion criteria:

- Too severely effected
- Referred for CBT assessment
- Unable to attend clinical sessions

Eligibility assessed for mood and anxiety interview

Inclusion criteria:

- Age 12 to 17

Exclusion criteria:

- Unable to complete diagnostic interview

Eligible and
accepted

Not eligible or declined

Not eligible or declined

Participate in MAGENTA only

Eligible and
consented

Not eligible or declined

Participate in MAGENTA including KSADS

Eligibility assessed for mood and anxiety study

Inclusion criteria:

- Age 12 to 18
- CFS/ME diagnosis

Exclusion criteria:

- Unable to complete questionnaires
- Unable to complete diagnostic interview

Eligible and
consented

Complete KSADS & RCADS

Data from SCAS, HADS, RCADS and interview analysed

**References**

1. Crawley E, Sterne JA. Association between school absence and physical function in paediatric chronic fatigue syndrome/myalgic encephalopathy. *Archives of disease in childhood.* 2009;94(10):752-756.

2. Crawley E, Hunt L, Stallard P. Anxiety in children with CFS/ME. *European child & adolescent psychiatry.* 2009;18(11):683-689.

3. Bould H, Collin SM, Lewis G, Rimes KA, Crawley E. Depression in paediatric chronic fatigue syndrome. *Archives of disease in childhood.* 2013;98(6):425-428.

4. Collin S, Nuevo R, van de Putte EM, Nijhof SL, Crawley E. Chronic fatigue syndrome (CFS) or myalgic encephalomyelitis (ME) is different in children compared to in adults: a study of UK and Dutch clinical cohorts. *BMJ open.* 2015;5(10):e008830.

5. Crawley E, Gaunt DM, Garfield K, et al. Clinical and cost-effectiveness of the Lightning Process in addition to specialist medical care for paediatric chronic fatigue syndrome: randomised controlled trial. *Archives of disease in childhood.* 2017.

6. Brigden A, Beasant L, Hollingworth W, et al. Managed Activity Graded Exercise iN Teenagers and pre-Adolescents (MAGENTA) feasibility randomised controlled trial: study protocol. *BMJ open.* 2016;6(7):e011255.
